# Improvement in Cognitive Status and Depressive Symptoms Three Months after Cataract Surgery

**DOI:** 10.22088/cjim.9.4.386

**Published:** 2018

**Authors:** Farzan Kheirkhah, GholamAbbas Roustaei, Elham Mohebbi Abivardi, Angela Hamidia, Sakineh Javadian Kutenai

**Affiliations:** 1 Social determinant of Health Research Center, Health Research Institute, Babol University of Medical Sciences, Babol, Iran; 2 Department of Psychiatry Babol University of Medical Sciences, Babol, Iran; 3 Department of Ophthalmology Babol University of Medical Sciences, Babol, Iran.; 4 Student Research Committee, Babol University of Medical Sciences, Babol, Iran.

**Keywords:** Depression, Cognition, Cataract Removal, Visual Acuity, Mental status and Dementia tests

## Abstract

**Background:**

Cataract induced vision impairment can lead to loss of older people’s independence and self-esteem and limit their daily activities. Moreover it has comorbid cognitive impairment and depression. Cataract surgery may be one way to attenuate these comorbidities. The aim of this study was to compare pre-operative and postoperative depressive symptoms and cognitive impairments of patients who underwent cataract surgery.

**Methods:**

This study was a before - after clinical trial. Participants completed the following validated surveys one day before and again three months after surgery. Dependent variables were preoperative to postoperative within-patient difference in Geriatric Depression Scale (GDS) and Mini-Mental State Examination (MMSE) scores. Independent variable was improvement of visual acuity.

**Results:**

Mean age was 71.77±8.08, 54% were females. Mean postoperative visual acuity improvement was 0.7720±0.1758, mean GDS score difference was -1.49±1.72 and mean MMSE score difference was 0.28±0.88. Postoperative improvement of visual acuity, GDS and MMSE scores were statistically significant (p=0.001). The mean visual acuity improvement in the participants with age over 80 years was lower than the younger subjects; while improvement in MMSE scores in this age group was significantly higher than them. There were no significant relationship between visual acuity, GDS and MMSE scores before and after surgery based on BMI and educational level.

**Conclusions:**

It was concluded that cataract surgery is effective for relieving depressive symptoms in the elderly. Improved visual acuity at older ages has far more effects on reducing cognitive impairment.

As people age, they may not only lose their mobility and independence but also their eyesight, limiting what they can and cannot do for entertainment and social life. Cataract is one of the main causes of a decrease in visual acuity in elderly patients and thereby a decrease in visual deprivation is related to quality of life (1). Protein aggregation within the lens due to aging is one of the main causes of cataract (2). In this study, we will be focusing only on senile cataract, and the term cataract will refer to a senile cataract. An estimated 20.5 million people over the age of 40 in America have cataract in either eye and is estimated to rise to 30.1 million by 2020 (3). Cataract-induced vision impairment can lead to a decrease in quality of life for elderly, difficulties in daily activities, and loss of independence and self-esteem (4).

Cataract-induced visual impairment can have a number of negative effects, particularly in the independence of an older individual. The presence of medical problems (e.g., diabetes, pulmonary disease, heart disease, and arthritis) is associated with an increased comorbidity with depression (5). The prevalence of depression among elderly people with visual impairment is about 29% (6). Visual impairment is a risk factor for depression because it prevents daily normal activities (7). On the other hand, cataract and cognitive impairment are both considered age-related health problems because they demonstrate a higher prevalence with advancing age (8). Despite the demographic overlap of cataract and cognitive impairment, their relationship has remained a neglected area of research. Understanding this relationship is important, however. As cataract is the leading cause of reversible blindness, cataract surgery may be one strategy for decreasing the burden of cognitive impairment. This study focuses on preoperative to postoperative improvement in depressive symptoms and cognitive status as a consequence of a successful cataract surgery. 

## Methods

 This study was a before - after clinical trial. In this study, 250 patients were enrolled from patients (≥ 60 years) who were diagnosed with bilateral cataract and primarily candidates for unilateral surgery of the worse eye between October 2016 to January 2017 at the Ayatollah Rouhani Hospital, Babol. With a 95% confidence interval, a 80% power and σ = 4 for Geriatric Depression Scale (GDS) and Mini-Mental State Examination (MMSE) scores to find one unit difference in these indices after surgery, The following formula was estimated to be 126 samples, and 250 samples were enrolled to increase the test power.

$n=\frac{{\left(\mathrm{Z1}-\frac{a}{2}+\mathrm{Z1}-\beta \right)}^{2}*\sigma^{2}}{d^{2}}$

, Z_1 - _α2=1.96_, _Z_1_ – β = 0.84, n = $\frac{({1.96+0.84)}^{2}*4^{2}}{1^{2}}=126$

The diagnosis of cataract was done through a clinical examination with slit lamp by single ophthalmologist (one of authors). The study was approved by the Ethics Committee of Babol University of Medical Sciences with code 524. The study was carried out in accordance with the declaration of Helsinki. Eligible subjects provided written informed consent, after receiving a complete description of the study. All participants who fully volunteered in this process, however, could leave the study at any time. 

Subjects were not paid for their participation. The inclusion criteria were bilateral senile cataract, decreased vision and patient’s desire for surgery; and the exclusion criteria were the other causes of decreased vision including glaucoma, age-related macular degeneration, uveitis, maculopathies, optic neuropathies, retinal vascular disorders and so on. 

The visual acuity of the patients was evaluated one week before and two weeks after phacoemulsification and monofocal foldable intraocular lens implantation. The surgeries were done by a single ophthalmologist (one of authors) under topical aesthesia.

 They were asked to respond to the MMSE, GDS and demographic questionnaires including sex, age, educational level, occupation, height, weight, drug use, FBS and HbA1c, one day before the surgery and three months afterwards. All data were collected by a senior psychiatry resident (one of authors). The GDS is a valid 15-item survey measuring symptoms of depression; possible score range is 0-15, higher score reflects more depressive symptoms, and a score of ≥ 6 is a positive screening for depression. The GDS was chosen for this study because it measures psychological symptoms of depression and not somatic symptoms which could be confounded by cataract-related symptoms. There are many brief cognitive assessments that are considered valid, reliable, and sensitive tests to screen for dementia. Of these, the MMSE is used most extensively in both the clinical and research settings to measure cognitive impairment (9). It tests five distinct cognitive domains: orientation, immediate memory, attention/ concentration, delayed recall, and language. The MMSE includes vision-independent and vision-dependent test items. Visual acuity of participants was assessed using the Snellen E chart. The Snellen E chart measures visual acuity based on letters set at certain ratios, and the 20/20 line is considered normal visual acuity. The measured visual acuities were converted to decimal equivalent for the convenience of statistical evaluation. They were assessed for best corrected vision at their initial appointment. Participants were assessed again two weeks postoperatively. 

One way ANOVA, chi-square and paired-samples t-test were used to assess some aspects of the visual related quality of life, which consisted of a rating of depression, the patient's cognitive status and visual acuity before and after cataract surgery. It should be noted that all statistical analyses were done by SPSS (IBM SPSS Statistics version 23). 

## Results

54 of 250 patients lost their third month follow-up. 196 remaining participants were aged between 60 and 92 years (mean and sd 71.77±8.08). Majority of the participants were females (n=100, 51%), did not have any education (n=143, 72.9%) and without diabetes (n=149, 76%). According to normal GDS score ≤ 5, majority of the participants (n=143, 73%) had some degree of depression. GDS scores of participants before surgery were between one and 12 with the mean and sd of 6.56±2.24. Three months after surgery, GDS scores were between zero and 12 with the mean and sd of 5.07±2.45. GDS scores were changed in the range of zero to six with the mean and sd of -1.49±1.72 after cataract remoral. According to normal MMSE score ≥ 23 (10), minority of the participants (n=43, 21.9%) had some degree of cognitive impairment. Before the surgery, MMSE score of participants was between eight and 29 with the mean and sd of 22.96±3.73. Three months after the surgery, MMSE scores were measured between nine and 29 with the mean and sd of 23.25±3.44. MMSE scores were changed in the range of -1 to five with the mean and sd of 0.28±0.88 after cataract surgery.

Visual acuity of participants before surgery was between 0.0052 and 0.4 with the mean and sd of 0.1133±0.0827. After the surgery, visual acuity of participants was in the range of 0.05-1 with the mean and sd of 0.8853±0.1964. The improvement of vision by cataract surgery was in the range of 0.04-0.99 with the mean and sd of 0.7720±0.1758. The mean improvement of vision among non-diabetic participants (0.8061±0.1621) was higher than the diabetic ones (0.6637±0.1752) (p<0.001). While, MMSE and GDS score changes were not related to diabetes.

**Table 1:** Comparison of mean visual acuity, GDS and MMSE score differences before and after cataract surgery

**Parameter**	**Preopeative ** **mean± SD**	**Postoperative** **mean±SD**	**difference ** **mean±SD**	**P-value**	**95% CI**
Visual acuity	0.1133±0.0827	0.8853±0.1964	0.7720±0.1785	.000	[0.7472, 0.7967]
GDS* score	6.56±2.24	5.07±2.45	-1.49±1.72	.000	[-1.73, -1.25]
MMSE** score	22.96±3.73	23.25±3.44	0.28±0.88	.000	[0.16, 0.41]

* GDS: Geriatric Depression Scale

** MMSE: Mini-Mental State Examination

In table 1, the paired- samples t-test was conducted to compare the mean visual acuity, GDS and MMSE scores before and after the cataract removal. There was a significant increase of mean visual acuity after the surgery than before the surgery. Also there was a significant improvement of mean GDS score postoperatively than preopevatively. Improvement of postopevatively MMSE score than preopevative was the same as GDS. 

According to the results of one way ANOVA for analysis of visual acuity improvement, MMSE and GDS scores in three age groups are presented in table 2, it can be seen that there is a statistically significant difference between the groups in the visual acuity improvement and MMSE improvement. There was no significant difference between the mean GDS changes in the three age groups. Scheffe post hoc test was used to track the difference between the groups. The results of this test showed that the mean visual acuity improvement in the participants with age over 80 years was lower than the other two groups; while improvement in the MMSE scores in this age group was significantly higher than the other two groups. Therefore, it can be concluded that an increase in the visual acuity at older ages has far greater effects on the improvement of cognitive impairment. The participants were divided into four groups based on BMI, underweight with BMI <18.50, normal 18.50-24.99, overweight 25.00-29.99 and obese ≥30. They compared with one way ANOVA test and there was no significant relationship between the visual acuity (F(3,192)= 0.661, p=0.577), GDS scores (F(3,192)= 1.726, p=0.163) and MMSE scores (F(3,192)= 1.485, p=0.22) before and after the surgery. The subjects were also compared with based on with one way ANOVA according to literacy, the results showed that pre oprative and postoperative visual acuity (F(2,193)= 0.865, p=0.423), GDS score (F(2,193)= 0.314, p=0.731) and MMSE score differences (F(2,193)= 0.864, p=0.423) had no statistically significant association with educational level.

**Figure 1:**
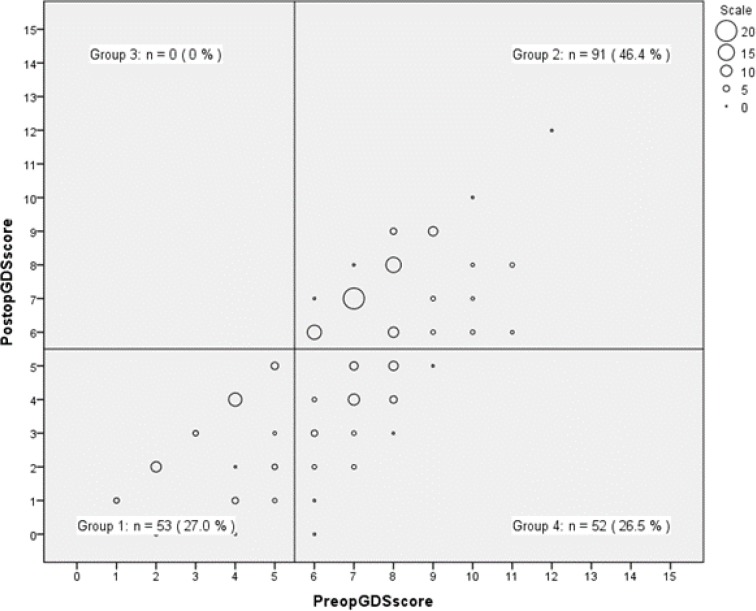
Categorization of participants based on preoprative and postoperative GDS scores

As demonstrated in figure 1, considering GDS score ≤5 as normal, the subjects were divided into four distinct groups: Group one (n=53, 27%) with normal preoperative and postoperative GDS scores, group two (n=91, 46.4%) with abnormal preoperative and postoperative GDS scores, group three (n=0) with normal preoperative and abnormal postoperative GDS scores and the intended one, group four (n=52, 26.5%) with abnormal preoperative and normal postoperative GDS scores. The score of group four score surpassed the normal level postoperatively, though it seems that the surgery was the most beneficial for this group. The relationship between pre and postoperative normal and abnormal GDS scores was evaluated with chi-square test. The difference between the groups was statistically significant (X^2^(1, n=196) = 62.96, p=.000).

**Table 2:** Comparison of mean visual acuity, GDS and MMSE score differences before and after cataract surgery according to the age group

Age group		difference visual acuity mean±SD	difference GDS* score mean±SD	difference MMSE** score mean±SD
60-69 (n = 82)		0.7936±0.1545	-1.43±1.73	0.06±0.39
70-79 (n = 75)		0.8066±0.1343	-1.55±1.82	0.2±0.7
≥ 80 (n = 39)		0.6600±0.2374	-1.51±1.54	0.92±1.46
ANOVA	F (2, 193)	11.013	0.098	15.307
	p-value	.000	0.907	.000
Scheffe	p-value	.000		.000
	95 % CIs	[-0.21, -0.05] [-0,23, -0.06]		[0.47, 1.25] [0.32, 1.12]

* GDS: Geriatric Depression Scale

** MSE: Mini-Mental State Examination

**Figure 2:**
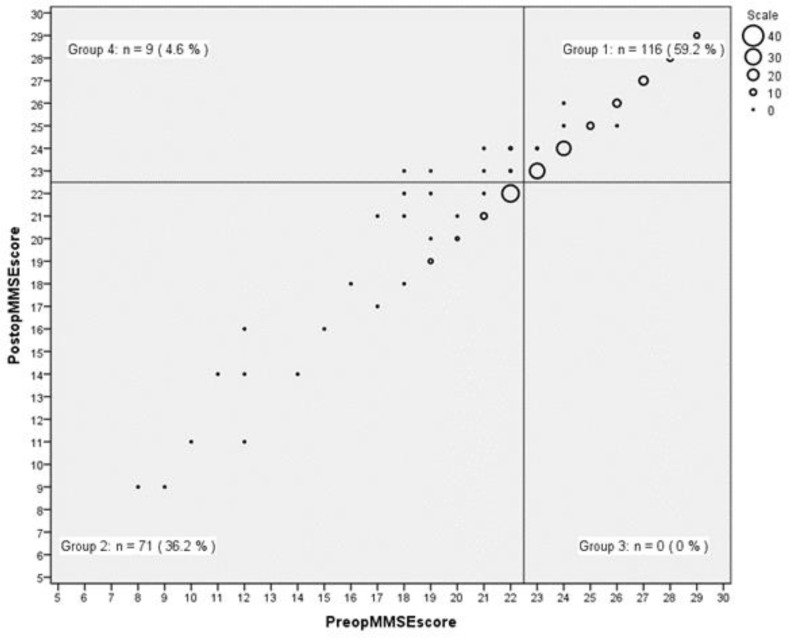
Categorization of participants based on preoprative and postoperative MMSE scores

The subjects, as shown in figure 2, according to their preoprative and postoperative MMSE scores, considering score ≥ 23 as normal were, divided into four distinct groups: Group one (n=116, 59.2%) with normal preoprative and postoperative MMSE scores, group two (n=71, 36.2%) with abnormal pre and postoperative MMSE scores, group three (n=0) with normal preoprative and abnormal postoperative MMSE scores and the intended one, group four (n=9, 4.6%) with abnormal preoprative and normal postoperative MMSE scores. Group four gained normal scores after the operation that they did not have previously, though probably they had the most benefit from the surgery. The relationship between normal and abnormal MMSE scores before and after the surgery was investigated with chi-square test. The difference between the groups was statistically significant (X^2^(1, n = 196) = 161.43, P=.000).

## Discussion

This study aimed to investigate the impact of cataract surgery on depressive symptoms and cognitive status in cataract patients undergoing surgery. It was hypothesized that the participants will exhibit a great decline in depressive symptoms, great increase in visual acuity and improvement in cognitive status. Consistent with the hypothesis, cataract surgery reduced depressive symptoms in our elderly patients (-1.42±1.72 changes in GDS score postoperatively). Few studies have examined cataract surgery’s effect on depression. Furthermore, results from the previous studies are inconsistent. McGwin et al. found that depressive symptoms did not change significantly from the baseline to the follow-up in those who underwent surgery and did not (11). In contrast, Ishii et al. claimed that depressive symptoms had statistically significant decrease postoperatively (12). Earlier, Fagerström had found a significant increase in visual acuity and decrease in depressive symptoms following the surgery (13).

We also found that visual acuity and depression were significantly correlated. The current findings are consistent with some of those previous studies demonstrating an increase in visual acuity and significant change in depressive symptoms following the surgery. In our study, the MMSE score of participants improved significantly (0.28±0.88) after the surgery; this change indicated that cataract surgery has had positive effect on the cognitive status of the elderly. Given the strong evidence for the negative effect of visual impairment upon cognitive status, in a patient whose poor vision can be attributed to cataract, surgical intervention may not only improve their vision, but also improve their mental status. Tamura et al. compared patients with cognitive impairment and cataract who had surgery with those who did not have surgery. 

They found that the operative group experienced a significant improvement in the revised Hasagawa Dementia Scale score after surgery, whereas the scores for the control group remained roughly the same (14). A few studies noted that the normal patients without cognitive impairment had an improvement in the MMSE scores after the cataract surgery, although there was a difference in the follow-up time: one observed an increase by six months postoperatively (15), whereas, others reported improvements sooner at two months (16). Jefferis et al. reported that the patients aged 75 years and older with bilateral cataract had a significant improvement in revised Addenbrooke’s Cognitive Examination (ACE-R) scores one year after the cataract surgery. Their results showed a significant improvement not only in total ACE-R scores but also in vision independent items scores (17). 

A longitudinal study by Hall et al. provided evidence contradicted this theory. Their three groups consisted of patients without cataract, patients with cataract who were candidates to have surgery, and patients with cataract who refused surgery; the patients without cataract did not have a change in Mattis Organic Mental Syndrome Screening Examination scores (4), which were consistent with the aforementioned studies. Nonetheless, the patients with cataract, regardless of whether the cataract surgery was performed or not, had significant improvement in their scores at one-year follow-up. Therefore, this improvement in tests scores cannot be explained by cataract surgery or improved visual function. Rather, they might be a result of learning from repeated cognitive tests. Similarly, other studies reported no difference in six cognitive tests in a population of women with or without previous cataract surgery (18), nor in postoperative patients with bilateral cataract versus non-operative controls (19).

As mentioned above, reports supporting the hypothesis that cataract surgery itself improves cognition are gradually increasing. Nevertheless, there is still insufficient evidence for cognitive improvement after the cataract surgery.

It is important to note that this study had no control group. There are other limitations in the present study. We had only one follow-up for tests after three months. Having multiple follow-up tests over a longer period of time may allow researchers to investigate the progression. 

Besides, the participants in the current study were fairly homogenous. Having conducted the present study from the population of some nearby cities, most of those who grew up in the area with similar values had fairly similar levels of education. In a heterogeneous sample for example, some people may be very explicit about their depressive symptoms, while other people may be embarrassed and their responses might be affected by their desire to not show weakness or need for help.

Our study had important implications for the comprehensive preoperative and postoperative evaluation of cataract surgery patients. It demonstrated improvements in mental well-being after the surgery among older people with cataract. Our findings showed that after the cataract surgery, depressive symptoms and cognitive status significantly improved.

**Key Messages:** Our study had important implications for the comprehensive preoprative and postoperative evaluation of cataract surgery patients. It demonstrated improvements in mental well-being after the surgery among older people with cataract. Our findings showed that after the cataract surgery, depressive symptoms and cognitive status significantly improved.
